# Hedgehog signalling in allograft vasculopathy: a new therapeutic target?

**DOI:** 10.1016/j.tips.2023.05.005

**Published:** 2023-06-07

**Authors:** Jasmine Rowell, Ching-In Lau, Diana C. Yánez, Eden Zhang, Tessa Crompton

**Affiliations:** 1 UCL Great Ormond Street Institute of Child Health, London, UK

## Abstract

Allograft vasculopathy (AV) leads to chronic rejection of organ transplants, but its causes are obscure. New research from the Jane-Wit laboratory showed that Sonic Hedgehog (SHH) signalling from damaged graft endothelium drives vasculopathy by promoting proinflammatory cytokine production and NLRP3-inflammasome activation in alloreactive CD4^+^PTCH1^hi^PD-1^hi^T memory cells, offering new diagnostic and therapeutic strategies.

SHH is essential during embryonic development, and as a morphogen it can signal for distinct outcomes dependent on the strength and duration of signal transduction [1]. After birth it regulates tissue homeostasis and repair, and is upregulated in tissues following injury or induction of inflammation where it signals to tissue-resident and recruited immune and inflammatory cells, and may have immunoregulatory or proinflammatory actions [2]. The canonical Hedgehog (Hh) signalling pathway is initiated by binding of SHH – or other Hh ligands: Indian Hedgehog (IHH) and Desert Hedgehog (DHH) – to their receptor, Patched1 (PTCH1), thereby allowing the signal transducer Smoothened (SMO) to be phosphorylated and enter the primary cilium to trigger downstream events. At the end of the pathway, the transcription factors GLI1, GLI2, and GLI3 regulate expression of Hh target genes through a consensus DNA binding motif. The pathway has multiple positive and negative feedback mechanisms, and PTCH1, itself an Hh target gene, can function to sequester Hh proteins. Noncanonical Hh pathway activation has also been described, including SMO-independent and PTCH1-independent signalling, and Hh signals can be transduced in T cells, which lack primary cilia [1,2].

AV is a major cause of chronic allograft rejection which has been extensively studied in heart transplant patients. In these patients, cardiac allograft vasculopathy (CAV) is the leading cause of death or retransplantation in the long term, and patients with CAV may be asymptomatic or show nonspecific symptoms, so CAV often presents as sudden death [3]. Thus, a better understanding of the causes of AV and new strategies for its diagnosis and prevention are urgently needed. The underlying mechanism that leads to CAV is not well understood, but it is believed to be immune-mediated, where alloreactive T cells drive a T helper1-type (Interferon γ, IFN-γ) response to the graft endothelial cells (ECs), leading to chronic inflammation, tissue damage, and ultimately organ failure [4].

An exciting recent study from the Jane-Wit laboratory sheds new light on the mechanisms leading to AV in chronic rejection of solid-organ transplants, demonstrating a role for SHH secreted from damaged graft endothelial tissue in signalling to alloreactive T cells to drive chronic vascular inflammation [5].

In the new study, humanised mouse models and mixed cell cultures were used to investigate the mechanisms that lead to alloreactive T cell responses against graft ECs [5]. The initial observation that SHH expression is upregulated in graft ECs when vascular damage occurs came from comparison of patient biopsies from renal and heart transplant recipients who experienced delayed graft function as a result of vascular damage, compared with control biopsies. The impact of SHH signalling on **allogeneic T cell responses** (see Glossary) and vascular inflammation were then investigated. *In vitro* treatment with a SMO agonist (SAG) together with CD3 ligation, but not SAG treatment alone, induced high cell-surface PTCH1 and programmed death 1 (PD-1) expression in CD4^+−^ CD45RO^+^ **memory T cells (T**_**mem**_**)** purified from peripheral blood. In coculture experiments with allogeneic ECs subjected to **ischaemia–reperfusion injury (IRI)**, CD4^+^PTCH1^hi^PD1^hi^T_mem_ cells were preferentially expanded by SHH secreted by ECs. SHH also enhanced **type I effector responses**, and expression of the chemokine receptor **C-C motif chemokine receptor 2 (CCR2)**, which enables homing to peripheral tissues, indicating that this CD4^+^PTCH1-^hi^PD1^hi^T_mem_ population could potentially migrate to the damaged graft *in vivo*. Their expansion was abrogated by pharmacological inhibitors of SMO, but less so by GLI inhibitors.

*In vivo* experiments in humanised mice – in which IRI-treated human artery segments were cografted with allogeneic human lymphocytes as a model for AV – showed that PTCH1^hi^T_mem_ cells were attenuated by treatment with pharmacological SMO inhibitors or GLI inhibitors, but severity of AV and strength of the type I response were more strongly inhibited by SMO inhibition, and adoptive transfer of different T_mem_ populations, defined by levels of cell-surface PTCH1, confirmed the contribution of the PTCH1^hi^T_mem_ population in AV.

Further investigation of the mechanism of type I T cell responses showed that SAG together with anti-CD3 treatment induced the **inflammasome** protein NLRP3 in PTCH1^hi^T_mem_ but that this was an indirect effect mediated by IFN-γ, whereas in EC:T-cell cocultures pharmacological inhibition of NLRP3 reduced the frequency of IFN-γ^+^PTCH^hi^T_mem_, an effect that was rescued by exogenous IL-18. The importance of NLRP3 downstream of SHH signalling in the allogenic T cell response was confirmed in the animal model, where SAG treatment was shown to exacerbate AV pathology, but cotreatment with an NLRP3 inhibitor together with SAG reduced AV pathology and serum IFN-γ.

In the final part of the study, the T cell autonomous role of the inflammasome-activating zinc finger protein ZFYVE21 was assessed downstream of SHH signalling. ZFYVE21 had previously been identified as a proinflammatory protein that leads to nuclear factor κB (NF-κB) activation in ECs in vascular inflammation [6].

In PTCH1^hi^T_mem_ cells *ZFYVE21* expression was induced in response to SAG and anti-CD3 treatment, and this induction was more strongly reduced by SMO inhibition than by GLI inhibition. Conversely, modulation of *ZFYVE21* expression showed that ZFYVE21 was required and sufficient for the expansion of the PTCH1^hi^PD-1^hi^CCR2^+^T_mem_ population, but did not directly prime NLRP3 inflammasomes. Experiments in the AV humanised mouse model confirmed that ZFYVE21 expression increased type I T cell reponses and exacerbated AV pathology to induce chronic inflammation.

Taken together, this series of experiments contributes significantly to our understanding of the mechanisms of AV, and identifies several molecules and pathways that could be targeted therapeutically (Figure 1). Lowering SHH signalling – either by treatment with neutralising antibodies against SHH or by pharmacological SMO inhibition – could be used to reduce the allogeneic T cell response to damaged ECs in allografts. Pharmacological SMO inhibitors are already licensed for the treatment of some cancers, so the Hh pathway is an attractive therapeutic target [7]. In mouse models of lung and skin inflammation, SMO inhibition reduced SHH expression in the damaged tissue [8,9], which would have additional benefits for AV. Additionally, the *in vivo* experiments demonstrated that pharmacological NLRP3 inhibition reduced AV pathology, and it may also have broader effects on other inflammatory cell types. Neutralisation of IL-18 or development of pharmacological ZFYVE21 inhibitors might also be effective. PD-1 agonism to inhibit the PTCH1^hi^PD-1^hi^T_mem_ population could also be considered.

The identification of this new mechanism for AV may also have diagnostic benefits, as patient blood could be screened for levels of SHH, IL-18, and circulating PTCH1^hi^PD-1^hi^T_mem_; this may be particularly beneficial for heart transplant patients in whom CAV can develop asymptomatically.

SHH signalling exacerbated the T cell-driven pathology of AV and increased expression of the proinflammatory cytokines IFN-γ and IL-18, but it acted in an IFN-γ-dependent manner rather than by driving inflammation independent of **cytokine signalling**. Tissue-derived SHH signalling has wide-ranging context-dependent pro- and anti-inflammatory effects in T cells [2]. Consistent with its role in amplifying the type I response in T_mem_ cells, it has previously been shown to enhance TH2 and regulatory T cell differentiation of naive CD4 T cells when activated in an appropriate cytokine milieu, but was unable to drive their differentiation in the absence of cytokine signalling [2,8,10]. Given the context dependency of the outcome of SHH signalling to T cells [2], the diverse effects of pharmacological SMO inhibition in mouse models of inflammation [8,9], and the fact that SHH can signal for distinct outcomes dependent on signal strength/duration, the use of SMO inhibitors for treatment of AV in solid organ recipients will require careful evaluation.

## Figures and Tables

**Figure 1.**
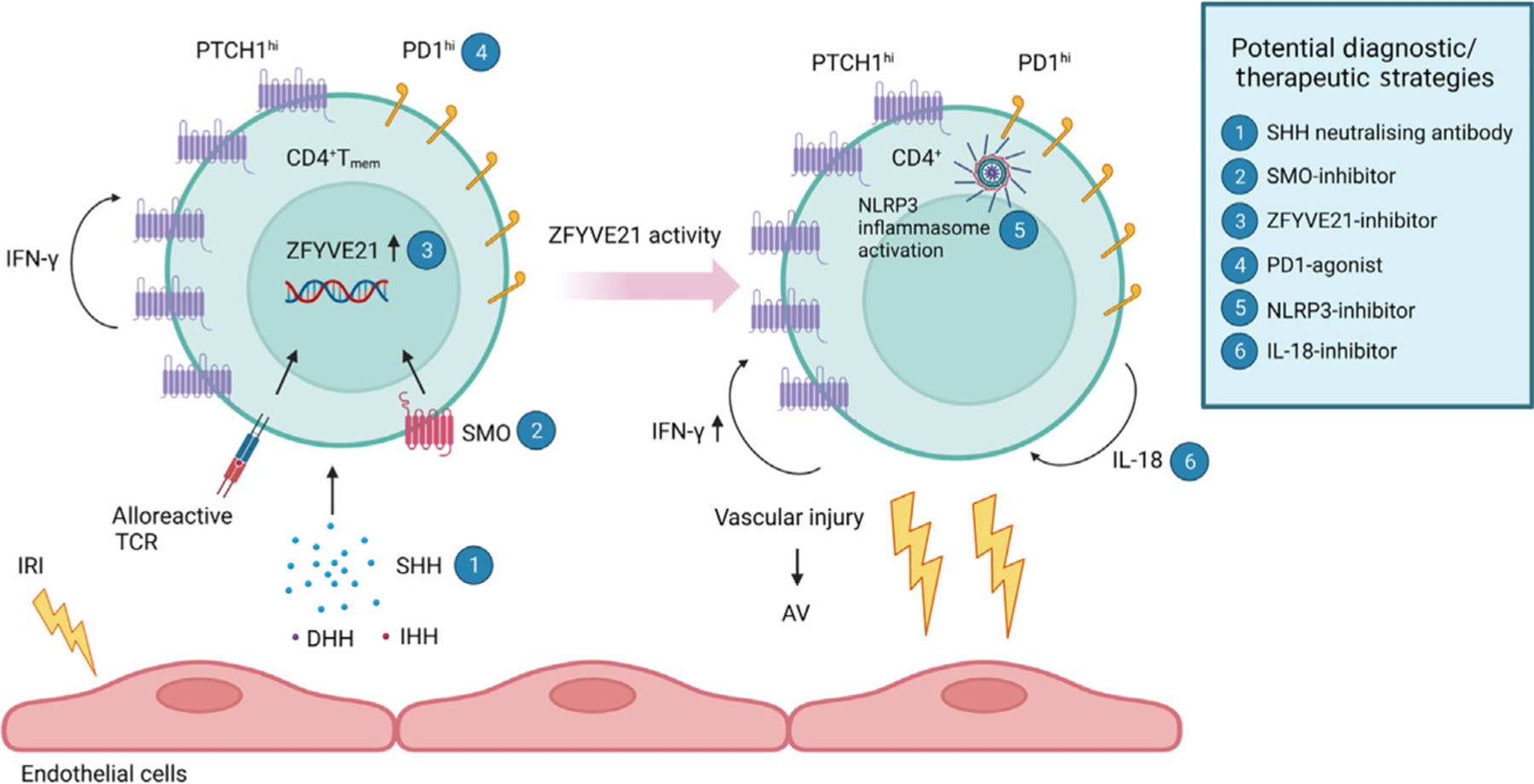
Mechanism of allograft vasculopathy (AV). The cartoon illustrates the newly described mechanism of AV [1]. Ischaemia–reperfusion injury (IRI) leads to Sonic Hedgehog (SHH) secretion by endothelial cells which promotes type I interferon γ (IFN-γ) responses in alloreactive CD4^+^PTCH1^hi^PD1^hi^T_mem_ cells. SHH activates Smoothened (SMO) signalling which leads to induction of zinc finger protein *ZFYVE21*. ZFYVE21 activates NLRP3 inflammasomes causing enhanced IFN-γ expression and interleukin 18 (IL-18) secretion, further driving the alloreactive response and leading to AV. The cartoon highlights novel possible therapeutic or diagnostic strategies for AV (numbered). Abbreviations: DHH, Desert Hedgehog; IHH, Indian Hedgehog; PD1, programmed death 1 protein; PTCH1, Patched1; TCR, T cell receptor. Figure created with BioRender.

## Glossary

Allogeneic T cell response: a powerful immune response that causes allograft rejection. It is a response against non-self and is usually polyclonal, involving interaction of the TCR with non-self MHC molecules.
C-C motif chemokine receptor 2 (CCR2): the receptor for the chemokine CCL2 (C-C motif chemokine ligand 2), and its signalling enables cells to home to tissues that express CCL2.
Cytokine signalling: signalling mediated by secreted proteins called cytokines that signal to immune cells and inflammatory cells to activate or regulate their functions.
Inflammasome: a cellular component that assembles in response to cellular damage or microbial infection, that when activated mediates caspase-1 activation and secretion of the proinflammatory cytokines IL-1β and IL-18.
Ischaemia–reperfusion injury (IRI): the exacerbation of cellular dysfunction, cell death, and tissue damage that occurs when blood flow is restored to previously ischaemic tissues. Ischaemia occurs when there is a decrease in blood supply to tissues, leading to decreases in oxygen and nutrients.
Memory T cells (T_mem_): a population of T cells that persists after having previously been exposed to an antigenic stimulation, such as an infection. On re-exposure they mount a more vigourous response to the antigen.
Type I effector response: a T cell response that is driven primarily by IFN-γ and by T helper 1 effector cells.
